# Dietary *Lycium barbarum* Polysaccharide Supplementation Modulates Serum Metabolomic Profiles and Gut Microbiota in Felines

**DOI:** 10.3390/ani16172765

**Published:** 2026-09-03

**Authors:** Xiao Zhang, Hua Yang, Xinda Liu, Weipeng Tian, Lei Pu, Liang Hong, Renjie Qin, Zhicheng Ning, Jianbin Zhang

**Affiliations:** 1 Tianjin Key Laboratory of Agricultural Animal Breeding and Healthy Husbandry, College of Animal Science and Veterinary Medicine, Tianjin Agricultural University, Tianjin 300392, China; youngxiao00@foxmail.com (X.Z.);; 2 Gambol Pet Food Group Co., Ltd., Liaocheng 252027, China

**Keywords:** *Lycium barbarum* polysaccharide, adult cats, serum metabolomics, gut microbiota, 16S rRNA sequencing, functional pet food, amino acid metabolism, lipid metabolism

## Abstract

Functional pet foods are increasingly being developed to support host metabolism and intestinal health. *Lycium barbarum* polysaccharide (LBP), a major bioactive component of goji berries, has been reported to possess antioxidant, anti-inflammatory, immunomodulatory, and gut microbiota-regulatory activities; however, its effects in domestic cats remain unclear. This study investigated the effects of dietary LBP supplementation on serum metabolomic profiles and gut microbiota in adult cats. Twenty-four healthy adult Chinese domestic cats (1.50 ± 0.50 years old; 3.70 ± 0.80 kg) were allocated to four groups using a body-weight-balanced procedure (n = 6 per group): a basal diet group (C) and basal diet supplemented with 0.2% (L), 0.3% (M), or 0.4% (H) LBP. After a 1-week adaptation period, the formal feeding trial lasted 8 weeks. Growth-related indices, health scores, hematological and serum biochemical parameters, untargeted serum metabolomics, and fecal 16S rRNA sequencing were evaluated. LBP supplementation did not significantly affect body weight, average daily feed intake, body condition score, fecal score, coat score, hematological indices, or serum biochemical parameters. Metabolomic analysis showed that LBP mainly affected lipid metabolism, unsaturated fatty acid biosynthesis, branched-chain amino acid metabolism, lysine degradation, and protein digestion and absorption-related pathways. Microbiota analysis showed changes in alpha diversity, community structure, and the relative abundance of taxa such as *Olsenella*, *Bifidobacterium*, *Catenibacterium*, *Peptoclostridium*, *Collinsella*, and *Clostridium*. Correlation analysis suggested potential associations between key bacterial genera and serum metabolites related to fatty acid and amino acid metabolism. Overall, dietary LBP altered serum metabolic signatures and gut microbial composition without causing adverse effects in adult cats, These exploratory findings require confirmation in larger feline studies and do not establish an optimal supplementation level.

## Simple Summary

*Lycium barbarum* polysaccharide (LBP) is a natural bioactive polysaccharide with potential nutritional and microbiota-modulating effects. In this exploratory study, healthy adult cats were fed diets supplemented with different levels of LBP for eight weeks. LBP supplementation did not adversely affect body weight, feed intake, clinical scores, hematological indices, or serum biochemical parameters. Serum metabolomics and 16S rRNA gene sequencing suggested changes in lipid- and amino-acid-related metabolic profiles and gut microbial composition. These preliminary findings support further investigation of LBP as a functional ingredient in adult feline diets.

## 1. Introduction

With the continuous expansion of pet ownership and increasing attention to pet health management, pet foods are evolving from products that merely meet basic nutritional requirements toward functional diets that provide nutritional support, health regulation, and disease-prevention potential. Dietary modulation is considered an important factor influencing the gastrointestinal microbiome and health status of cats and dogs [1]. Functional pet foods containing natural bioactive compounds, prebiotics, probiotics, and plant extracts have received growing attention because of their potential to maintain host health, modulate gut microbiota, and regulate immune function; dietary modulation of the gut microbiome in dogs and cats has also become an active research field [2]. Natural plant-derived bioactive ingredients are of particular interest because of their broad availability, relatively high safety, and multi-target regulatory properties.

*Lycium barbarum* polysaccharide (LBP) is one of the major active components of goji berries. Goji berries and their bioactive constituents have been reported to exert antioxidant, anti-inflammatory, and metabolic regulatory effects [3]. Previous studies have shown that LBP can influence immune function and alter gut microbial composition [4]. Increasing evidence suggests that interactions among LBP, intestinal microorganisms, and microbial metabolites may contribute to host health regulation [5]. LBP has also been described as having prebiotic-like properties and may influence intestinal microbiota and immune status [6]. In terms of metabolic regulation, animal-model studies have shown that LBP can modulate lipid metabolism and gut microbial composition [7]. However, evidence regarding the application of LBP in pet nutrition, particularly in cats, is still limited, and its effects on feline serum metabolism and gut microbiota remain unclear.

High-throughput sequencing and metabolomics provide useful approaches for understanding the mechanisms by which functional dietary ingredients act. Gut microbiota analysis based on 16S rRNA sequencing and untargeted mass spectrometry-based metabolomics are now widely used to investigate host–microbe interactions. The feline gut microbiota has a complex community structure and can be influenced by diet composition, functional ingredients, and host physiological status [8]. Dietary resistant starches and fibers can alter fecal microbiota and metabolomic profiles in healthy cats, indicating that the feline intestinal ecosystem is sensitive to nutritional interventions [9]. QIIME 2 has been widely used for reproducible and standardized microbiome data analysis [10], while metabolomics platforms such as MetaboAnalyst are commonly used for metabolite screening, pathway enrichment, and interpretation of multi-omics datasets [11]. Therefore, integrating gut microbiota profiling with serum metabolomics can provide a systematic view of the regulatory effects of LBP and support the selection of appropriate supplementation levels.

In the present study, healthy adult cats were fed basal diets supplemented with different levels of LBP. Serum untargeted metabolomics and fecal 16S rRNA sequencing were performed to evaluate the effects of LBP on host metabolic profiles and gut microbial structure. This study aimed to clarify the potential regulatory effects and dose–response characteristics of LBP in adult cats and to provide theoretical and experimental support for its use in functional cat food development.

## 2. Materials and Methods

### 2.1. Animals, Health Screening, and Management

Twenty-four healthy adult Chinese domestic cats were used in this study. Their mean age was 1.50 ± 0.50 years and their mean body weight was 3.70 ± 0.80 kg. Each dietary group included three males and three females, and all cats were intact. Before the trial, all cats underwent clinical health screening to ensure that they met the inclusion criteria. Health screening included general clinical examinations of mental status, appetite, body temperature, respiration, heart rate, skin and coat condition, and fecal appearance, combined with body weight, body condition score, and visual health assessment. All enrolled cats showed normal mental status, feed intake, and water intake, with no vomiting, diarrhea, skin disease, marked emaciation, obesity, or other obvious clinical abnormalities. Before the experimental diets were assigned, the cats were allocated to four groups using a body-weight-balanced procedure; no computer-generated random sequence was used.

Before the trial, all cats had completed routine vaccination and internal and external parasite control according to husbandry requirements. All cats had been maintained on the same diet before enrollment. During the 1-week adaptation period, all cats were uniformly fed the basal diet used in the formal experiment to minimize the influence of previous dietary exposure. During the formal trial, cats were housed individually with free access to water. The room temperature was maintained at 22–26 °C and relative humidity at 50–60%. Cages were cleaned daily and disinfected regularly. Mental status, feed intake, fecal output, and abnormal clinical signs were recorded daily.

### 2.2. Experimental Materials

#### 2.2.1. *Lycium barbarum* Polysaccharide

The LBP used in this study was purchased from Shaanxi Tianyun Biotechnology Co., Ltd. (Xi’an, China; batch number TY20250716; manufacture date 16 July 2025). According to the certificate of analysis supplied by the manufacturer, the product was a powdered commercial plant extract with a labeled polysaccharide content of 80%, and the measured polysaccharide content was 80.13%, as determined by ultraviolet spectrophotometry using glucose as the reference standard. According to additional information provided by the supplier, the product was prepared by water extraction followed by ethanol precipitation, filtration, concentration, and drying. The product also met the manufacturer’s specifications for sensory characteristics, particle size, identification, ash content (1.91%), loss on drying (2.01%), heavy metals, pesticide residues, and microbiological quality. During the trial, LBP was stored in a cool, dry, and dark environment according to the manufacturer’s instructions. Detailed purification conditions, molecular-weight distribution, monosaccharide composition, and structural characterization by HPLC, FTIR, or GPC/HPGPC were not available from the supplier and were not independently determined in the present study.

#### 2.2.2. Basal Diet

The basal diet was a commercial complete dry food formulated for adult cats. According to the manufacturer, the main ingredients included salmon (27%), fresh chicken, corn, beef bone meal, chicken oil, fish oil (3%), soybean meal, corn gluten meal, beet pulp (2%), carrot powder, green tea powder (0.1%), and flaxseed oil. All cats received the same batch of basal diet throughout the trial. The guaranteed nutrient composition declared by the manufacturer is presented in Table 1. These values were not independently measured in the present study.

### 2.3. Experimental Design

The 24 cats were allocated to four dietary groups using a body-weight-balanced allocation procedure, with six cats per group and one cat as the experimental unit. Each group included three males and three females, and all cats were intact. The control group (C) received the basal diet, whereas the L, M, and H groups received the basal diet supplemented at nominal levels of approximately 0.2%, 0.3%, and 0.4% LBP, respectively. No computer-generated random sequence was used.

Experimental diets were prepared weekly. For the L, M, and H treatments, 20, 30, or 40 g of LBP powder, respectively, was added to 10 kg of the pelleted basal diet. Each batch was manually mixed thoroughly until no visible LBP powder remained on the surface or at the bottom of the mixing container. The prepared diets were stored in sealed containers under dry conditions at room temperature, used within 7 days, and manually turned and remixed before each feeding to reduce settling. The uniformity of LBP distribution was not analytically verified.

### 2.4. Growth Performance and Health Scores

On days 1 and 56 of the formal trial, cats were weighed in the morning after fasting to record initial and final body weight. Daily feed intake was recorded throughout the trial to calculate average daily feed intake (ADFI). Body condition was evaluated using a 9-point body condition score (BCS) system [12]. Coat condition was evaluated using a 5-point scoring system based on gloss, softness, cleanliness, hair loss, and dander. Fecal consistency was evaluated weekly using a 7-point fecal scoring system. Each score type was assessed by one designated trained evaluator throughout the study, and all evaluators were blinded to dietary treatment allocation. The use of the same evaluator for each score type and predefined criteria was intended to reduce inter-observer variation.

### 2.5. Blood and Fecal Sample Collection

At the end of the trial, feed was withdrawn for 8 h before blood collection. Blood samples were collected from all cats at approximately 09:00 within a similar time window to minimize potential effects of feeding status and circadian variation. Approximately 5 mL of blood was collected from the cephalic vein of each cat and placed into plain tubes, EDTA-anticoagulant tubes, and heparin sodium-anticoagulant tubes. Blood in the plain tubes was centrifuged at 3000 r/min for 15 min to separate serum, which was stored at −80 °C for untargeted serum metabolomics. EDTA-anticoagulated samples were used for hematological analysis, and heparinized samples were used for biochemical analysis. Fresh fecal samples were collected aseptically at the end of the trial, snap-frozen in liquid nitrogen, and stored at −80 °C for 16S rRNA sequencing.

### 2.6. Measurements and Analytical Methods

#### 2.6.1. Hematological and Serum Biochemical Measurements

Hematological parameters were measured using an automatic veterinary hematology analyzer, and serum biochemical parameters were measured using an automatic biochemical analyzer according to the manufacturers’ instructions for the instruments and reagent kits.

#### 2.6.2. Untargeted Serum Metabolomics Workflow

Untargeted serum metabolomic profiling was performed by Novogene Co., Ltd. (Beijing, China). Serum samples stored at −80 °C were thawed on ice. An aliquot of 100 μL serum was mixed with 400 μL of 80% methanol, vortexed, incubated on ice for 5 min, and centrifuged at 15,000× *g* for 20 min at 4 °C. The supernatant was diluted with LC–MS-grade water to a final methanol concentration of 53%, followed by a second centrifugation at 15,000× *g* for 20 min at 4 °C. The resulting supernatant was collected for LC–MS/MS analysis. A pooled quality-control (QC) sample was prepared by mixing equal volumes of all experimental samples, and a blank sample containing 53% methanol was processed in parallel to remove background ions and monitor analytical stability. Serum preparation and LC–MS/MS procedures followed established large-scale metabolomic profiling practices and current recommendations for untargeted LC–MS workflows [13,14].

Chromatographic separation was performed using a Vanquish ultra-high-performance liquid chromatography system coupled to an Orbitrap Exploris 120 mass spectrometer (Thermo Fisher Scientific, Waltham, MA, USA). Samples were separated on a Hypersil Gold C18 column (100 mm in length × 2.1 mm internal diameter, with a 1.9 μm particle size) maintained at 40 °C at a flow rate of 0.2 mL/min. Mobile phase A was 0.1% formic acid in water and mobile phase B was methanol. The gradient program was as follows: 0–1.5 min, 98% A; 1.5–3 min, 98–15% A; 3–10 min, 15–0% A; 10–10.1 min, 0–98% A; and 10.1–12 min, 98% A.

The mass spectrometer was operated with electrospray ionization in both positive- and negative-ion modes over an m/z range of 100–1500. The spray voltage was 3.5 kV, the sheath gas flow rate was 35 psi, the auxiliary gas flow rate was 10 L/min, the capillary temperature was 320 °C, the S-lens RF level was 60, and the auxiliary gas heater temperature was 350 °C. Data-dependent MS/MS acquisition was used.

Raw data were converted to mzXML format using ProteoWizard 3.0. Peak extraction, quantification, retention-time correction, and alignment were performed using XCMS. Metabolite features were matched against the Novogene in-house high-quality MS/MS spectral database (NovoMetDB) using accurate mass, retention time, adduct information, and MS/MS spectra with a mass tolerance of 10 ppm. Features missing in more than 50% of samples were removed, and remaining missing values were imputed using the k-nearest-neighbor method. Blank samples were used to remove background ions, and compounds with a coefficient of variation greater than 30% in QC samples were excluded. Relative peak areas were normalized before statistical analysis. Metabolites were further annotated using the KEGG, HMDB, and LIPID MAPS databases. Principal component analysis and partial least-squares discriminant analysis were performed using metaX. Differential metabolites were screened using VIP > 1.0, *p* < 0.05, and FC > 1.2 or FC < 0.833, followed by KEGG pathway enrichment analysis. Because the M vs. C and H vs. C PLS-DA models had limited predictive performance, PLS-DA was treated as an exploratory visualization and was not used alone to establish differential metabolites.

#### 2.6.3. 16S rRNA Gene Sequencing Analysis of Gut Microbiota

Genomic DNA was extracted from fecal samples using a commercial DNA extraction kit. The V3–V4 region of the bacterial 16S rRNA gene was amplified using primers CCTAYGGGRBGCASCAG and GGACTACNNGGGTATCTAAT. Sequencing libraries were constructed and paired-end sequencing was performed on an Illumina NovaSeq platform. Raw reads were merged and quality-filtered, and chimeric sequences were removed. Amplicon sequence variants were inferred using DADA2 in QIIME 2, followed by taxonomic annotation, relative abundance analysis, alpha-diversity and beta-diversity analyses, and differential-taxon analysis. LEfSe was applied using an alpha value of 0.05 and an LDA score threshold of 4.0. This relatively stringent threshold was selected to retain taxa with larger discriminatory effect sizes and reduce the reporting of weak features. No microbiome functional prediction was included in the revised manuscript.

#### 2.6.4. Use of Generative Artificial Intelligence

ChatGPT (version 5.5, OpenAI, San Francisco, CA, USA) was used to assist with English-language polishing, the initial conceptual design of the graphical abstract, and the organization and verification of selected secondary calculations during manuscript revision. The tool was not used to generate experimental data or independently determine scientific conclusions. All AI-assisted calculations, text, and graphical suggestions were independently checked, critically reviewed, and revised by the authors, who take full responsibility for the accuracy and integrity of the final manuscript.

### 2.7. Statistical Analysis

Experimental data were analyzed using SPSS 27.0.1.0 software. Normality was assessed using the Shapiro–Wilk test, and homogeneity of variances was evaluated using Levene’s test. Variables satisfying both assumptions were analyzed using one-way analysis of variance (ANOVA), followed by Duncan’s multiple-comparison test when the overall group effect was significant. Normally distributed variables with unequal variances were analyzed using Welch’s ANOVA. Non-normally distributed variables and ordinal score data were analyzed using the Kruskal–Wallis test. Results are presented as mean ± standard deviation (SD), and *p* < 0.05 was considered statistically significant. For metabolomics, PCA and PLS-DA were used for exploratory multivariate analysis. PLS-DA model quality was summarized using R^2^Y and Q^2^Y, and permutation testing was used to assess overfitting. Differential metabolites were screened using VIP > 1.0, *p* < 0.05, and FC > 1.2 or FC < 0.833. Spearman rank correlations were calculated between the prespecified key bacterial genera and key serum metabolites using the 23 samples with matched microbiome and metabolomics data. Nominal significance was denoted by *p* < 0.05, *p* < 0.01, and *p* < 0.001. To account for multiple testing within this genus–metabolite correlation matrix, all correlation *p* values were adjusted using the Benjamini–Hochberg procedure. Associations with FDR q < 0.10 were considered exploratory FDR-supported trends rather than confirmatory findings. Correlations were interpreted as associations and not as evidence of causality. LEfSe was performed using an alpha level of 0.05 and an LDA score threshold of 4.0; this relatively stringent cutoff was selected to retain taxa with larger discriminatory effect sizes and reduce weak or potentially unstable features.

## 3. Results

### 3.1. Growth Performance and Health Status

As shown in Table 2, the initial body weights did not differ significantly among groups (*p* > 0.05), indicating that the groups were comparable at baseline. After eight weeks of feeding, final body weight, absolute body-weight change, percentage body-weight change, and ADFI were not significantly different among groups (*p* > 0.05). Body weight increased slightly in the L and H groups and decreased slightly in the M group, but these changes were not statistically significant. ADFI was similar among all groups, indicating that LBP supplementation did not markedly affect feed intake.

LBP supplementation at 0.2–0.4% did not significantly affect body condition score, fecal score, or coat score (*p* > 0.05). Fecal scores remained within the normal range and coat scores were generally stable, suggesting no obvious adverse effects on body condition, fecal characteristics, or coat status.

### 3.2. Hematological and Serum Biochemical Parameters

Compared with the control group, dietary supplementation with different levels of LBP had no significant effect on hematological parameters in adult cats (*p* > 0.05; Table 3). All parameters remained within the normal reference range for healthy cats. Similarly, serum biochemical parameters did not differ significantly among groups (*p* > 0.05; Table 4), indicating that the LBP supplementation levels used in this study did not adversely affect hepatic function, renal function, lipid metabolism, or protein metabolism as reflected by routine blood indices.

### 3.3. Untargeted Serum Metabolomic Analysis

#### 3.3.1. PLS-DA Model Evaluation

**Figure 1:**
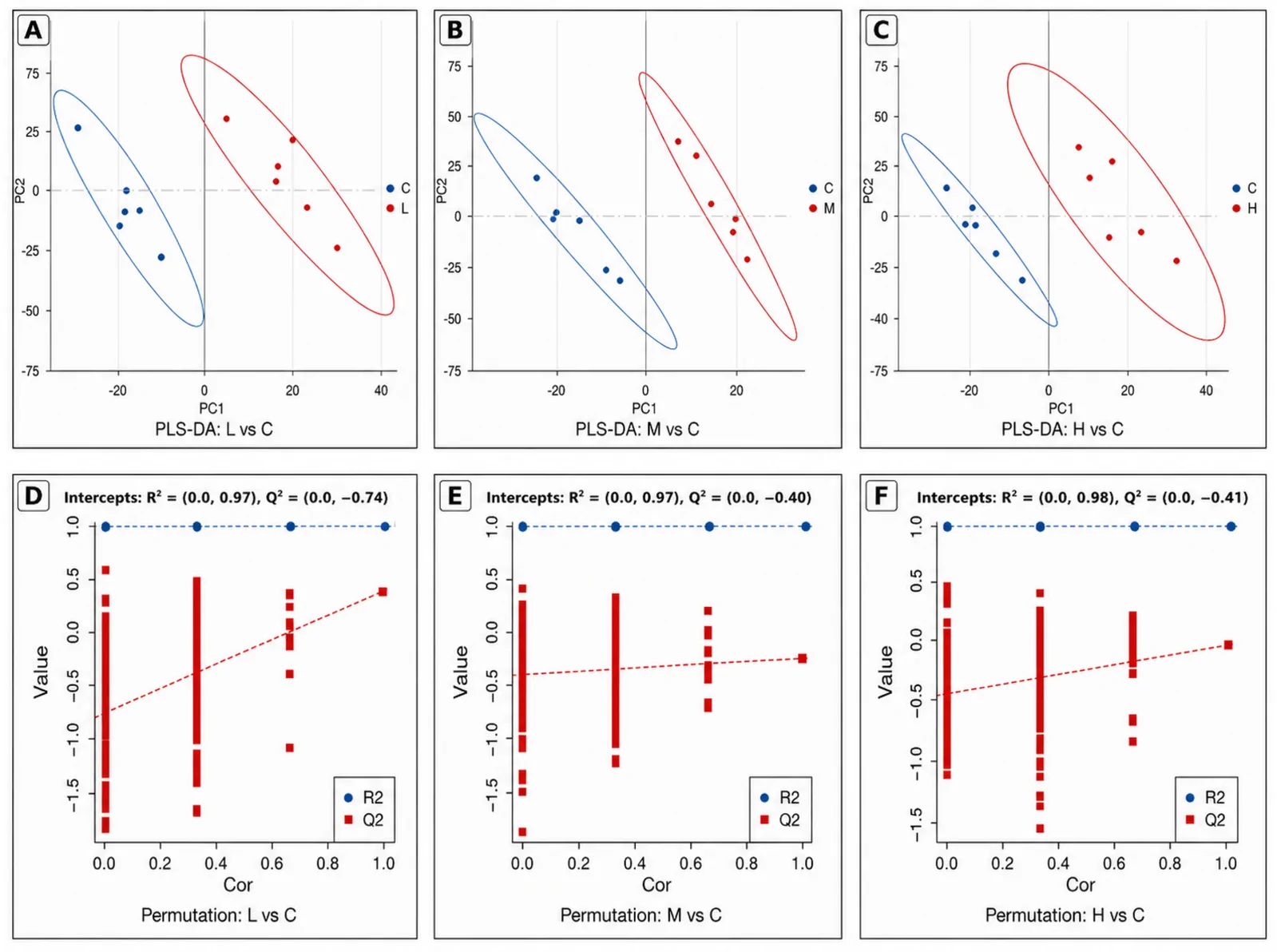
PLS-DA score plots and corresponding permutation tests of combined positive- and negative-ion serum metabolomic data. (**A**,**D**) L vs. C; (**B**,**E**) M vs. C; (**C**,**F**) H vs. C. The R^2^Y/Q^2^Y values were 0.98/0.40, 0.99/−0.26, and 0.97/−0.01, respectively. The corresponding R^2^/Q^2^ permutation-test intercepts were 0.97/−0.74, 0.97/−0.40, and 0.98/−0.41. The negative Q^2^Y values for M vs. C and H vs. C indicate limited predictive performance, and these models were interpreted cautiously.

To further explore metabolic differences between each LBP-supplemented group and the control group, supervised partial least-squares discriminant analysis (PLS-DA) was performed. The score plots showed apparent separation between groups; however, model performance differed among comparisons. The R^2^Y/Q^2^Y values were 0.98/0.40, 0.99/−0.26, and 0.97/−0.01 for L vs. C, M vs. C, and H vs. C, respectively. Thus, only the L vs. C model showed moderate predictive ability, whereas the M vs. C and H vs. C models had limited predictive performance. Permutation-test R^2^/Q^2^ intercepts were 0.97/−0.74, 0.97/−0.40, and 0.98/−0.41, respectively. Accordingly, PLS-DA was interpreted as exploratory and was not used alone to establish metabolic differences. Table 5 summarizes the model parameters, and Figure 1 shows the corresponding plots.

#### 3.3.2. Metabolite Class Composition

**Figure 2:**
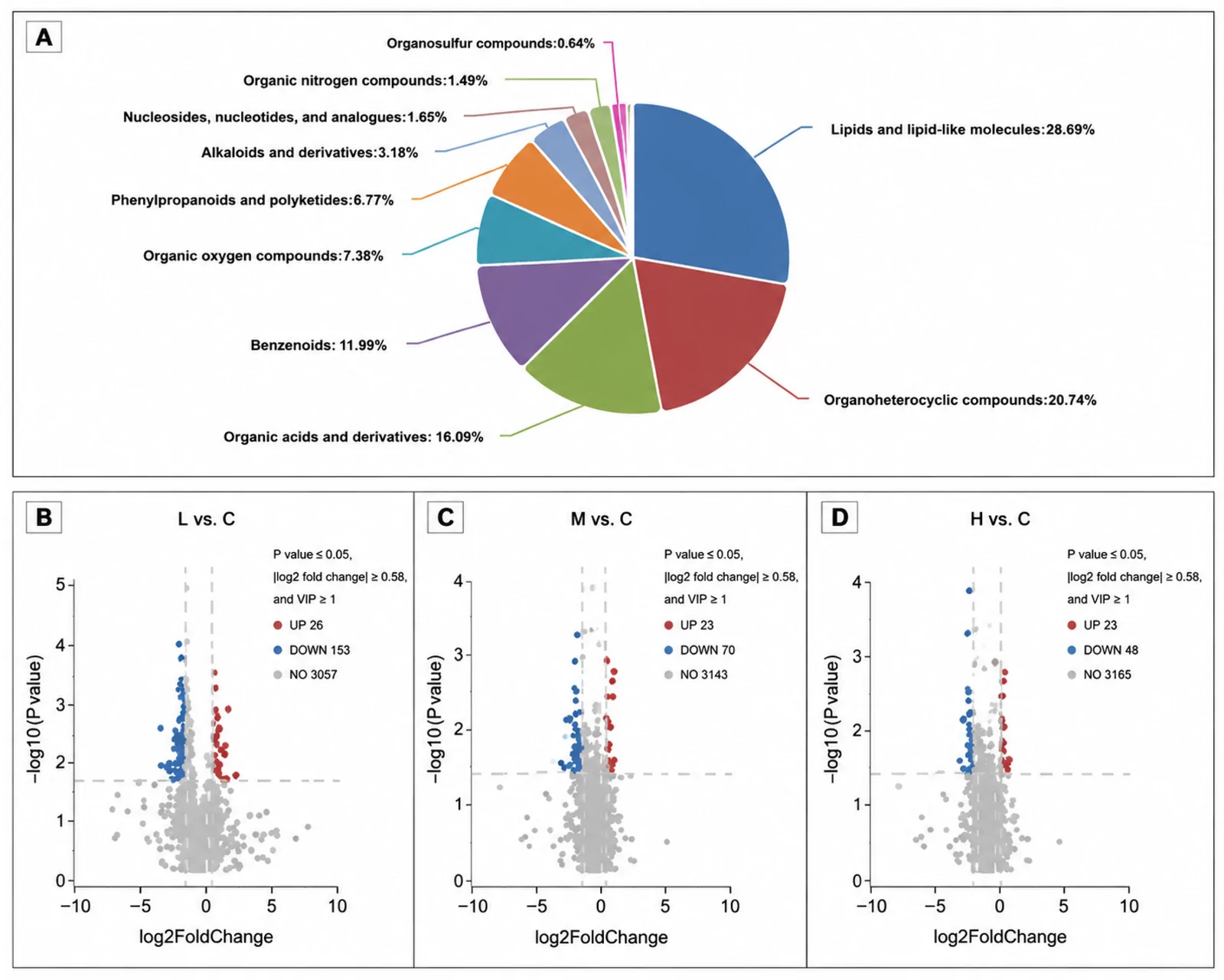
Serum metabolite class composition and differential metabolite screening. (**A**), The top 10 superclasses accounted for 98.62% of the annotated metabolites; the remaining 1.38% comprised minor superclasses and is not shown; (**B**–**D**), volcano plots for L vs. C, M vs. C, and H vs. C, respectively. Red dots indicate upregulated metabolites, blue dots indicate downregulated metabolites, and gray dots indicate non-significant metabolites. The screening criteria were VIP > 1.0, *p* ≤ 0.05, and |log2FC| ≥ 0.58.

The metabolite class composition showed that lipids and lipid-like molecules (28.69%), organoheterocyclic compounds (20.74%), and organic acids and derivatives (16.09%) were the predominant metabolite classes, together accounting for more than 65% of the annotated metabolites (Figure 2A). Benzenoids, organic oxygen compounds, phenylpropanoids and polyketides, and other classes also contributed to the serum metabolome, whereas organic nitrogen compounds and organosulfur compounds accounted for relatively small proportions. MetaboAnalyst-based approaches are commonly used for metabolomic data processing, visualization, and pathway interpretation [15].

#### 3.3.3. Differential Metabolite Screening

Differential metabolites were screened using VIP > 1.0, *p* < 0.05, and FC > 1.2 or FC < 0.833 as the criteria, and volcano plots were generated (Figure 2B–D). In the H vs. C comparison, 71 differential metabolites were identified, including 23 upregulated and 48 downregulated metabolites. In the M vs. C comparison, 93 differential metabolites were identified, including 23 upregulated and 70 downregulated metabolites. In the L vs. C comparison, 179 differential metabolites were identified, including 26 upregulated and 153 downregulated metabolites. Overall, each LBP-supplemented group showed differential metabolic responses, with downregulated metabolites predominating. The number of differential metabolites was highest in the L group, followed by the M and H groups.

#### 3.3.4. Venn Analysis of Differential Metabolites

**Figure 3:**
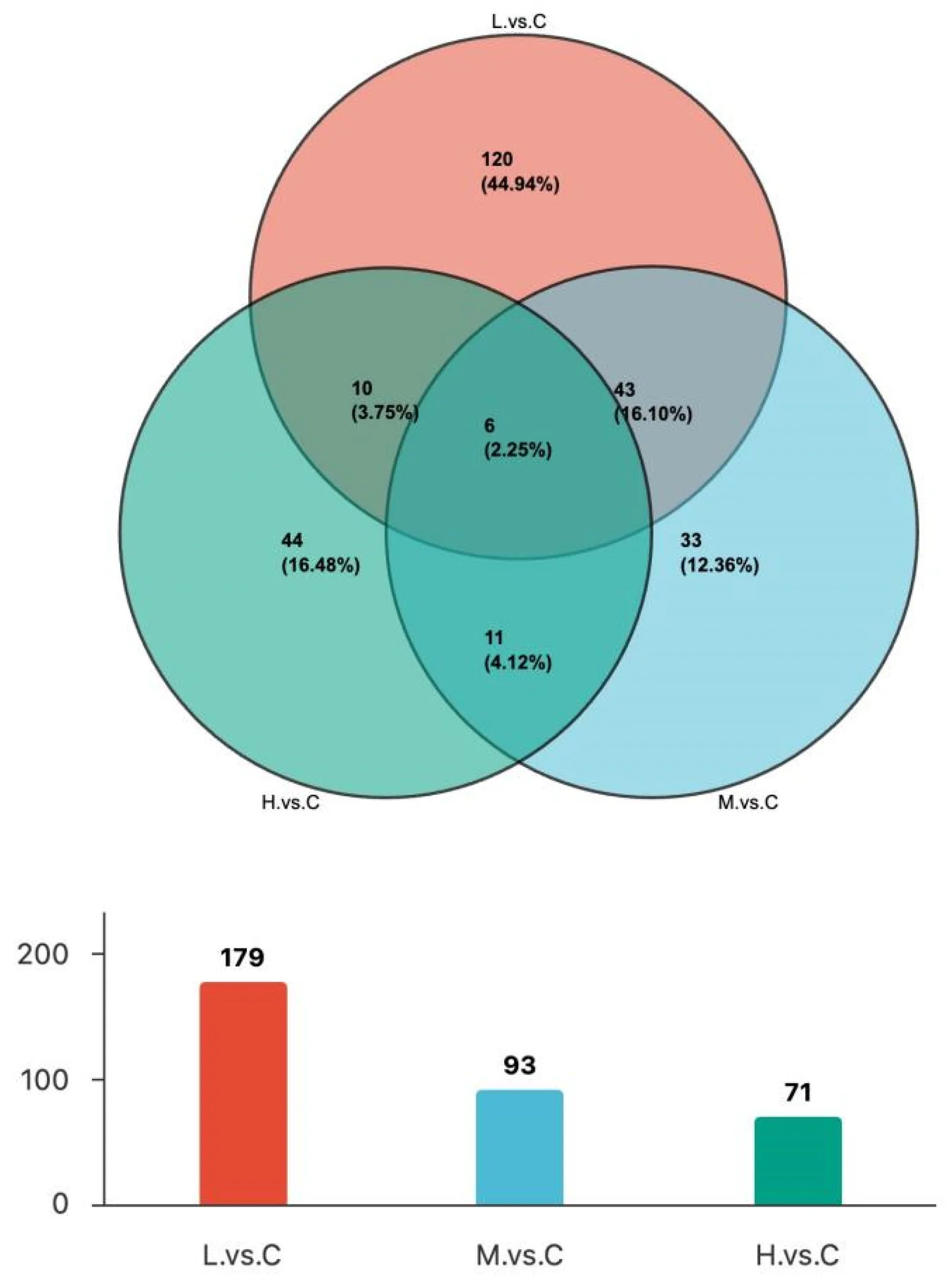
Venn diagram of differential metabolites identified in L vs. C, M vs. C, and H vs. C comparisons.

Venn analysis showed that the L group had the largest number of differential metabolites (179), followed by the M group (93) and the H group (71), indicating a tendency for the number of differential metabolites to decrease with increasing LBP dose. Six differential metabolites were shared among all three comparisons, suggesting that different LBP doses may have partially common metabolic targets. In addition, each comparison had unique differential metabolites: 120 in the L group, 33 in the M group, and 44 in the H group (Figure 3). The larger number of differential metabolites in the L group suggests a broader effect on serum metabolic profiles, whereas the H group showed fewer differential metabolites but pathway changes that were more concentrated in amino acid and protein-related metabolism. These findings indicate dose-specific effects of LBP on feline serum metabolism.

#### 3.3.5. KEGG Enrichment Analysis

**Figure 4:**
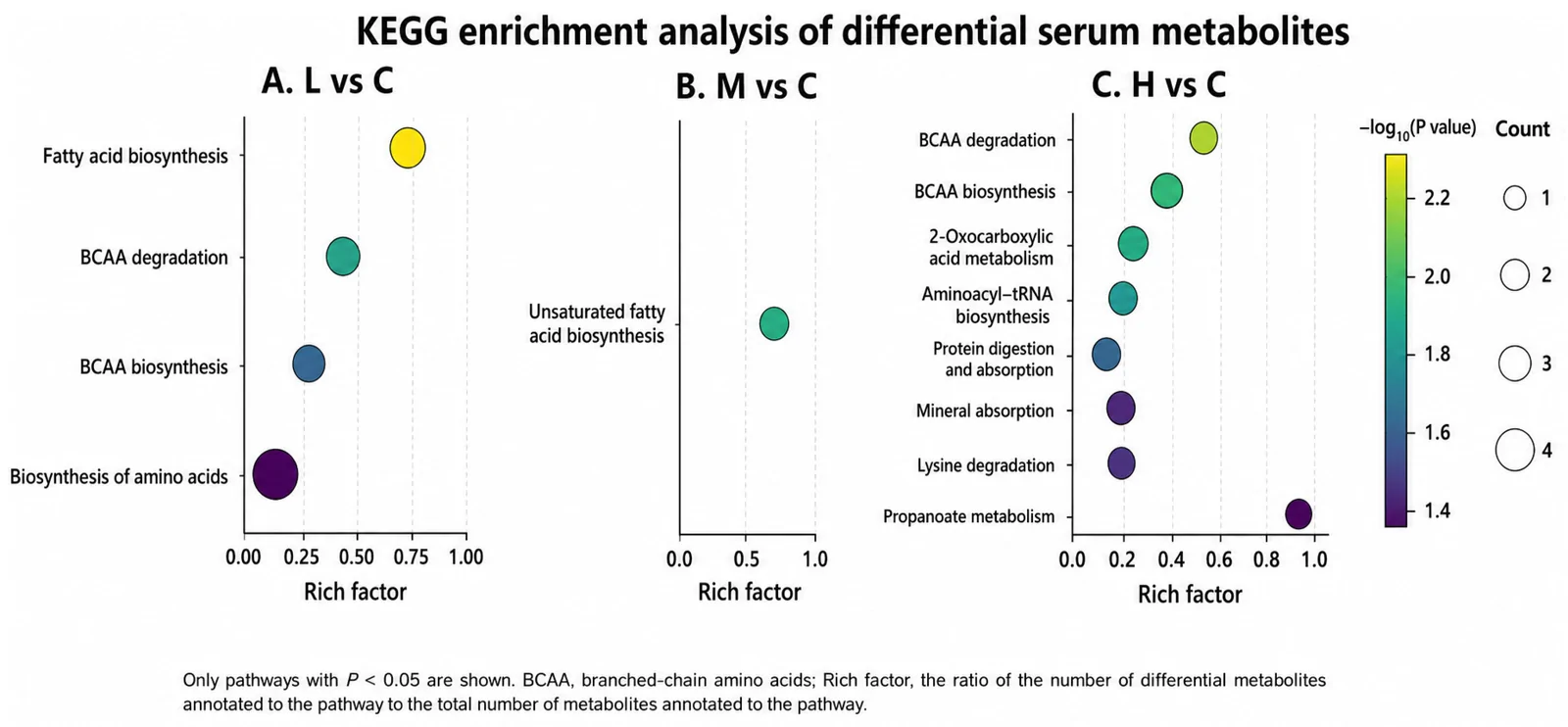
KEGG enrichment analysis of differential metabolites between LBP-supplemented groups and the control group. (**A**), L vs. C; (**B**), M vs. C; (**C**), H vs. C. The *x*-axis represents the rich factor (the ratio of differential metabolites mapped to a pathway to all identified metabolites annotated to that pathway), bubble size represents the hit count, and bubble color represents −log10(*p* value). Pathways were interpreted as exploratory where FDR-adjusted significance was not available. BCAA, branched-chain amino acids, including valine, leucine, and isoleucine.

KEGG enrichment analysis was performed for differential metabolites in each treatment group compared with the control group (Figure 4). In the L vs. C comparison, the differential metabolites were mainly enriched in fatty acid biosynthesis, branched-chain amino acid (BCAA) degradation, BCAA biosynthesis, and amino acid biosynthesis, suggesting that low-dose LBP may influence lipid and amino acid metabolism. In the M vs. C comparison, differential metabolites were significantly enriched in unsaturated fatty acid biosynthesis, indicating that medium-dose LBP may primarily modulate unsaturated fatty acid metabolism. In the H vs. C comparison, enriched pathways included BCAA degradation, BCAA biosynthesis, aminoacyl-tRNA biosynthesis, 2-oxocarboxylic acid metabolism, protein digestion and absorption, mineral absorption, lysine degradation, and propanoate metabolism, suggesting that high-dose LBP exerted more pronounced effects on amino acid and related organic acid metabolism.

Overall, low- and medium-dose LBP mainly affected lipid metabolism and part of amino acid metabolism, whereas high-dose LBP was associated with changes in protein digestion/absorption-related and amino acid metabolism pathways. These results suggest dose-associated differences in the metabolic effects of LBP in adult cats. The key differential metabolites are summarized in Table 6.

In the L vs. C comparison, key differential metabolites included palmitoleic acid, decanoic acid, octadecanoic acid, L-leucine, and L-valine, all of which were downregulated in the L group. This suggests that low-dose LBP may influence fatty acid biosynthesis and BCAA metabolism. In the M vs. C comparison, key metabolites were mainly related to unsaturated fatty acid metabolism; nervonic acid was downregulated and linoleic acid was upregulated in the M group. In the H vs. C comparison, key metabolites included H-Val-Val-OH, L-leucine, L-valine, L-lysine, L-pipecolic acid, linoleic acid, and 2-methylbutyric acid. Among these, H-Val-Val-OH, L-leucine, L-valine, L-lysine, and L-pipecolic acid were downregulated, whereas linoleic acid and 2-methylbutyric acid were upregulated, suggesting that high-dose LBP had stronger effects on protein digestion and absorption, BCAA metabolism, lysine degradation, and organic acid metabolism.

**Figure 5:**
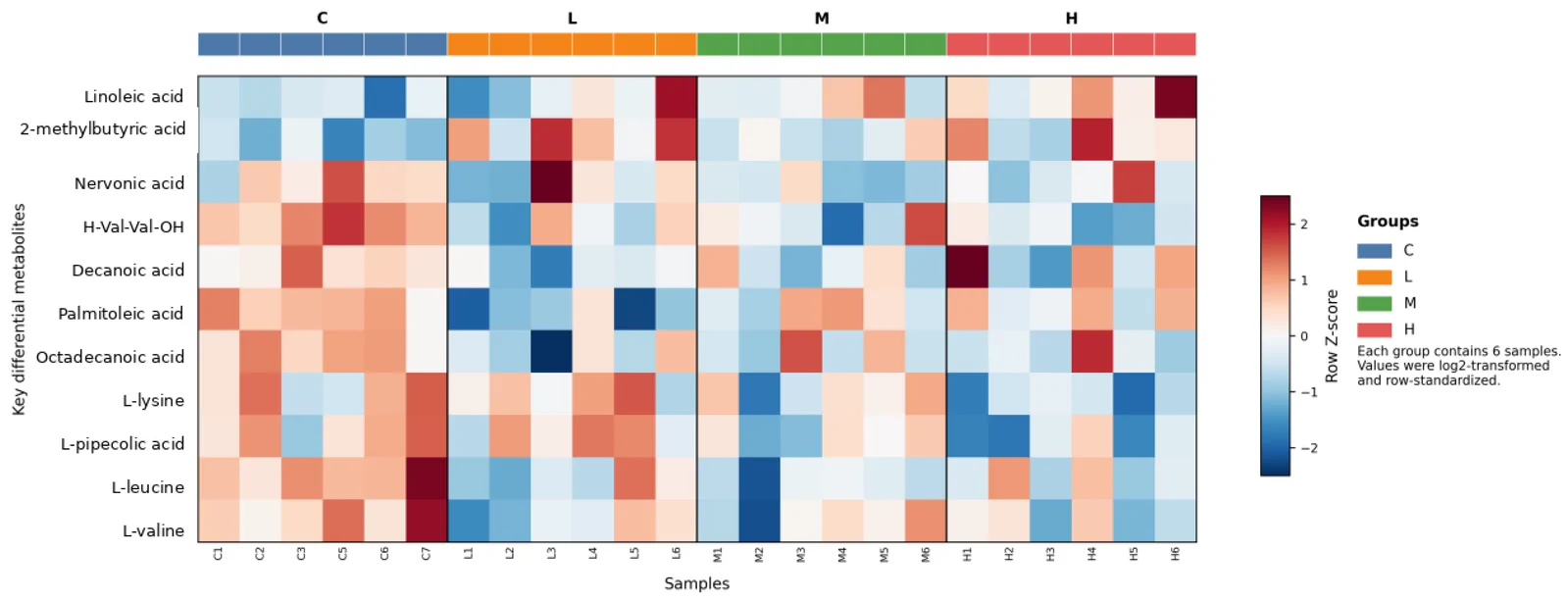
Hierarchical clustering heatmap of key differential metabolites after LBP intervention. Columns represent samples from the control group (C) and low-, medium-, and high-dose LBP groups (L, M, and H; n = 6 per group). Rows represent key differential metabolites. Colors indicate standardized relative abundance, with red indicating higher abundance and blue indicating lower abundance.

Hierarchical clustering based on these key metabolites showed distinct expression patterns among the C, L, M, and H groups (Figure 5). Fatty acid-related metabolites, including palmitoleic acid, decanoic acid, octadecanoic acid, nervonic acid, and linoleic acid, varied across LBP doses. BCAA- and lysine-related metabolites, including L-leucine, L-valine, L-lysine, and L-pipecolic acid, also showed group-dependent changes. These findings further suggest that LBP supplementation affects serum lipid and amino acid metabolic signatures in a dose-related manner.

### 3.4. Gut Microbiota Structure

#### 3.4.1. Rarefaction and Good′s Coverage Curves

Rarefaction and Good′s coverage curves were used to evaluate sequencing saturation and coverage. As the number of sequences increased, the observed features curves gradually reached a plateau at approximately 20,000 sequences, indicating that further sequencing would yield few additional features. Good′s coverage curves approached 1 (>0.99) at approximately 10,000 sequences, indicating that the vast majority of microbial taxa were effectively captured and that the sequencing depth was sufficient for subsequent diversity and community-structure analyses. The standardized microbiome data-processing framework was consistent with QIIME 2-based analytical principles [16], and high-resolution amplicon sequence inference was conceptually aligned with DADA2-based denoising methods [17].

#### 3.4.2. Alpha Diversity

**Figure 6:**
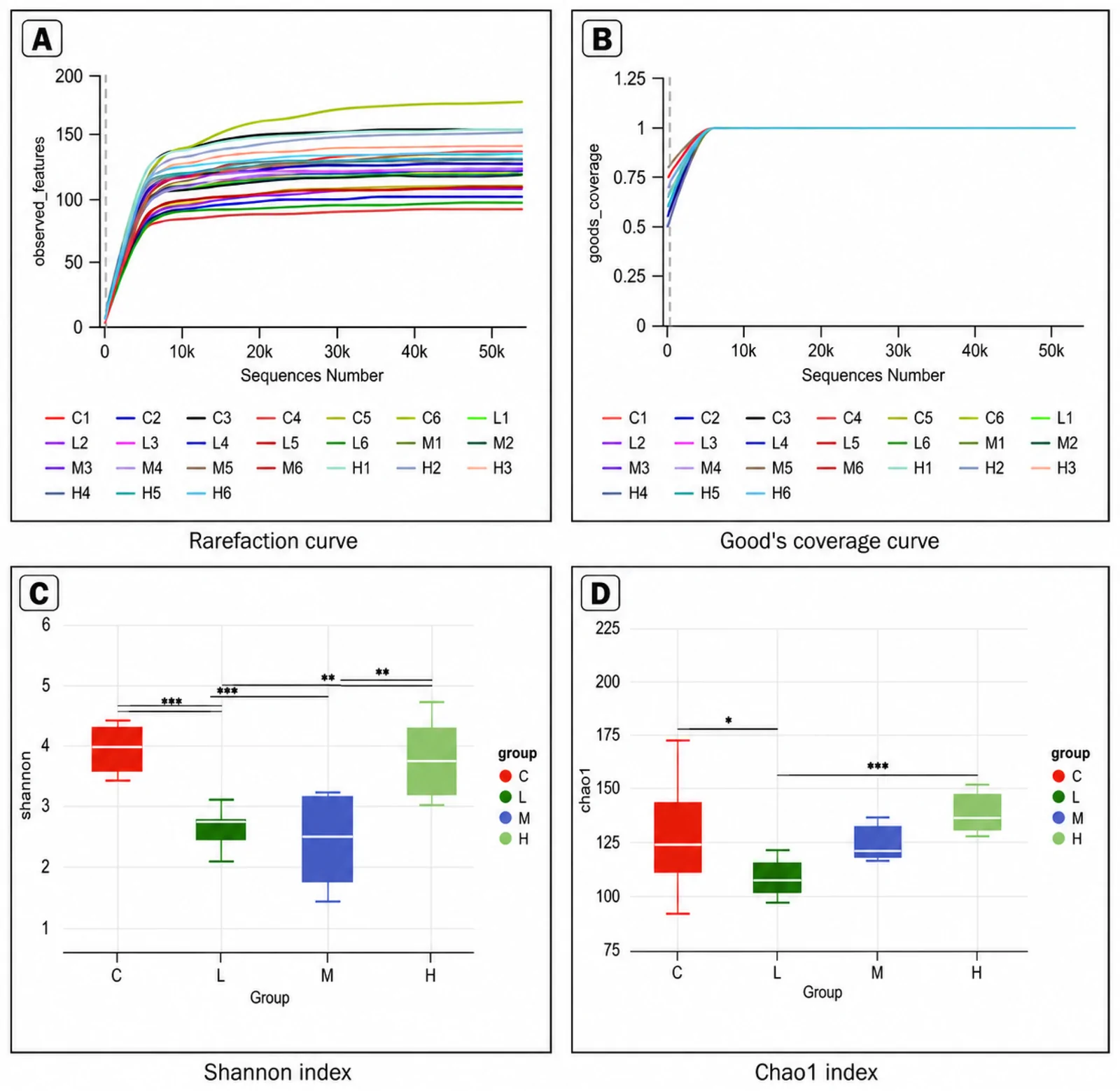
Sequencing depth and alpha diversity of gut microbiota. (**A**) rarefaction curve; (**B**) Good′s coverage curve; (**C**) Shannon index; (**D**) Chao1 index. Rarefaction and Good′s coverage curves show that sequencing depth was sufficient for downstream analysis. Alpha diversity results indicate that different levels of LBP supplementation affected gut microbial diversity and richness in adult cats. Asterisks indicate statistically significant differences between the groups connected by horizontal lines: * *p* < 0.05, ** *p* < 0.01, and *** *p* < 0.001.

The Kruskal–Wallis test was used to assess differences in alpha diversity among groups. The Shannon index reflects overall community diversity, considering both richness and evenness, whereas the Chao1 index mainly reflects species richness. Compared with the C group, the L group showed significantly lower Shannon and Chao1 indices (*p* < 0.05 or *p* < 0.01), suggesting that low-dose LBP reduced microbial diversity and richness. The M group showed partial recovery of both indices but remained lower than the control group. The H group showed further recovery; the Shannon index was no longer significantly different from that of the C group, and the Chao1 index was significantly higher than that of the L group. These results indicate dose-related differences in the effects of LBP on gut microbial diversity and richness (Figure 6).

#### 3.4.3. Beta Diversity

Principal coordinate analysis (PCoA) based on Bray–Curtis distances showed a clear separation tendency between the control group and the LBP-supplemented groups. ANOSIM analysis indicated significant differences in microbial community structure between C and L (R = 0.520, *p* = 0.002), C and M (R = 0.672, *p* = 0.005), and C and H (R = 0.270, *p* = 0.043) (Table 7), confirming that LBP supplementation significantly affected gut microbial community structure. The differences between the control group and the low- and medium-dose groups were highly significant, indicating that these two supplementation levels had stronger effects on community structure. The difference between L and M was not significant (R = 0.098, *p* = 0.195), suggesting a relatively high similarity between these two groups. LEfSe analysis was used to identify discriminant taxa, consistent with the biomarker discovery approach proposed for metagenomic data [18].

#### 3.4.4. Phylum- and Genus-Level Community Composition

**Figure 7:**
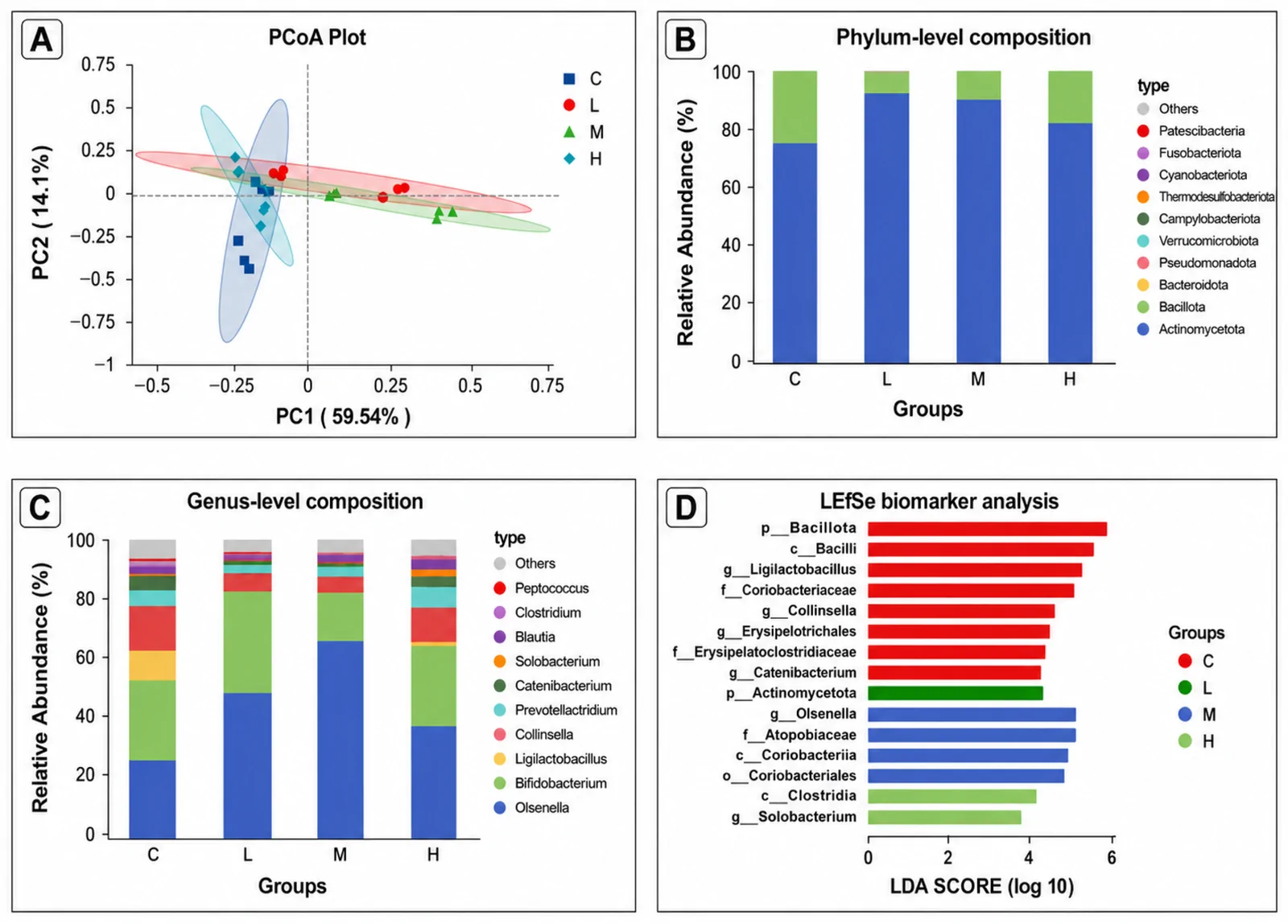
Gut microbiota beta diversity, community composition, and differential taxa. (**A**) PCoA plot based on microbial community structure; (**B**) phylum-level community composition; (**C**) genus-level community composition; (**D**) LEfSe analysis of differential taxa. PCoA results show a separation tendency among groups; community composition results indicate differences at the phylum and genus levels; LEfSe analysis identifies characteristic taxa in different groups.

To reveal the effects of different LBP doses on gut microbial composition, taxonomic composition was analyzed at the phylum and genus levels. At the phylum level, Actinomycetota and Bacillota were the dominant phyla in all groups and together accounted for more than 95% of the microbial community (Figure 7B). Compared with the C group, the L and M groups had higher relative abundance of Actinomycetota and lower abundance of Bacillota. In the H group, Actinomycetota decreased and Bacillota partially recovered, indicating a dose-related effect on dominant phyla.

At the genus level, *Olsenella* showed a pattern highly consistent with the overall trend of Actinomycetota; it increased markedly in the L and M groups and declined in the H group (Figure 7C). This suggests that *Olsenella* may be one of the core genera driving changes in Actinomycetota. *Bifidobacterium* was relatively abundant in the control group, decreased in the L and M groups, and partially recovered in the H group. In addition, the relative abundance of genera such as *Collinsella* and *Clostridium* varied among groups. These results indicate that different LBP levels changed gut microbial composition mainly through shifts in dominant phyla and their key genera.

#### 3.4.5. Identification of Key Differential Taxa

LEfSe analysis was performed with an LDA score threshold of 4.0 to identify key differential taxa (Figure 7D). The biomarkers in the C group mainly included Bacillota-related taxa, *Ligilactobacillus*, *Collinsella*, and *Catenibacterium*. The L group was characterized by Actinomycetota, whereas the M group was enriched in *Olsenella* and related Actinomycetota taxa, consistent with the phylum- and genus-level composition results. The H group was characterized by Clostridia and *Solobacterium*. These findings suggest that LBP supplementation regulated the gut microbial community in a dose-related manner. Although microbial functional prediction can be used for microbial function prediction [19], functional interpretations based on 16S rRNA data should be considered exploratory.

### 3.5. Correlation Between Gut Microbiota and Key Differential Metabolites

**Figure 8:**
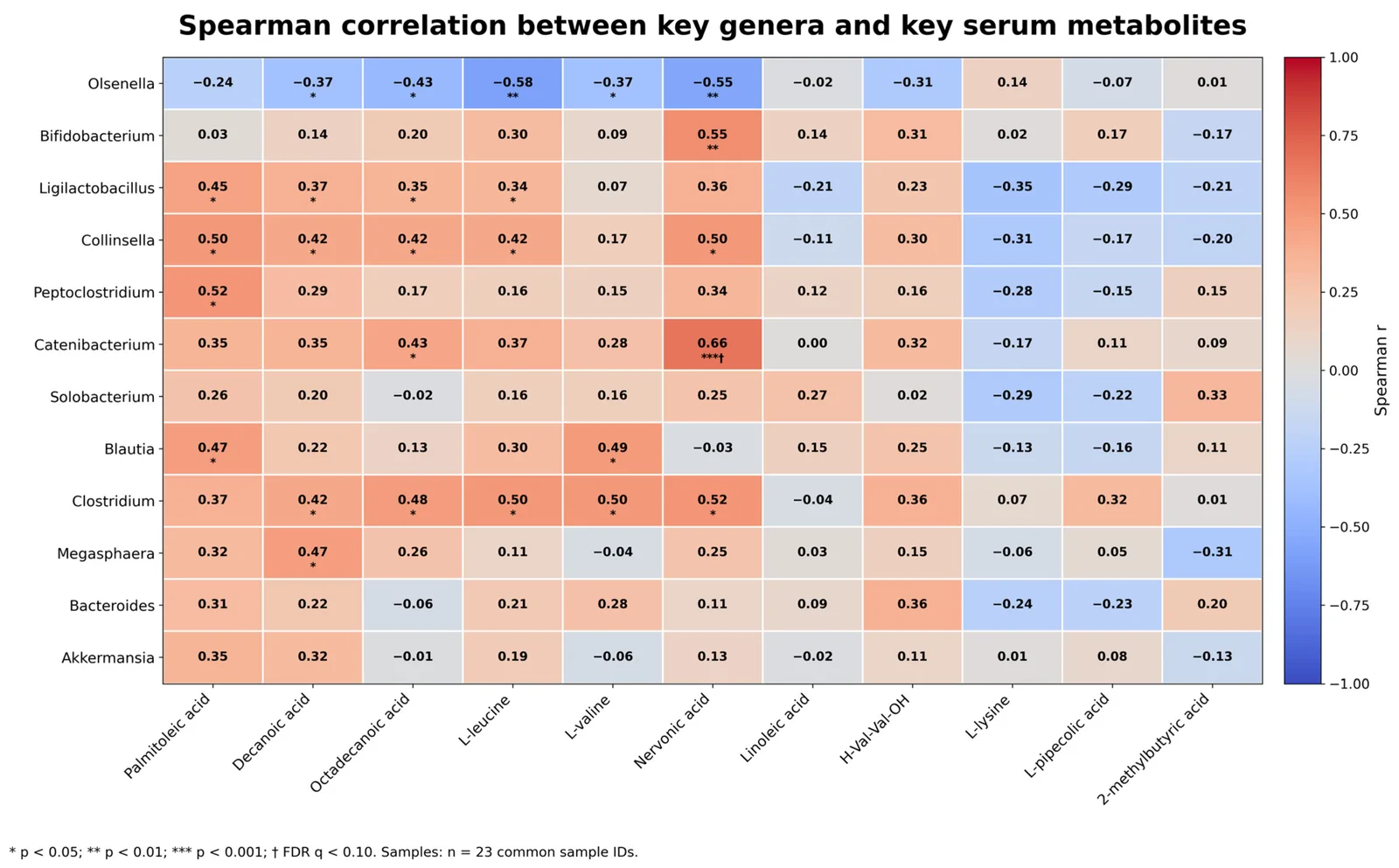
Spearman rank correlations between key bacterial genera and differential serum metabolites using 23 matched samples. Colors represent Spearman correlation coefficients. Asterisks denote nominal significance (* *p* < 0.05, ** *p* < 0.01, and *** *p* < 0.001), whereas the dagger (†) denotes an exploratory Benjamini–Hochberg FDR-supported association at q < 0.10. Correlations without a dagger did not meet the exploratory FDR threshold and should be interpreted as nominal associations.

Correlation analysis showed several nominal associations between differential genera and serum metabolites related to fatty acid and amino acid metabolism (Figure 8). *Catenibacterium* was positively correlated with nervonic acid (r = 0.66, *p* < 0.001) and remained an exploratory FDR-supported trend (q < 0.10). *Bifidobacterium* was positively correlated with nervonic acid (r = 0.55, *p* < 0.01), whereas *Collinsella* showed a nominal positive correlation with nervonic acid (r = 0.50, *p* < 0.05). *Peptoclostridium* was nominally positively correlated with palmitoleic acid (r = 0.52, *p* < 0.05), and *Collinsella* showed nominal positive associations with palmitoleic acid, decanoic acid, and octadecanoic acid. Except for the association marked with a dagger in Figure 8, these correlations did not meet the exploratory FDR threshold and were therefore interpreted cautiously.

For amino acid-related metabolites, *Olsenella* was negatively correlated with L-leucine (r = −0.58, *p* < 0.01) and nervonic acid (r = −0.55, *p* < 0.01), suggesting potential associations with amino acid and lipid metabolism. *Clostridium* was positively correlated with L-leucine, L-valine, nervonic acid, decanoic acid, and octadecanoic acid. *Blautia* was positively correlated with L-valine, and *Megasphaera* was positively correlated with decanoic acid. Overall, *Catenibacterium*, *Olsenella*, *Collinsella*, *Clostridium*, and *Peptoclostridium* were significantly associated with key metabolites such as nervonic acid, palmitoleic acid, decanoic acid, octadecanoic acid, L-leucine, and L-valine. These results suggest that LBP-induced changes in gut microbiota may be linked to alterations in serum lipid and amino acid metabolism.

## 4. Discussion

This study evaluated the effects of dietary LBP supplementation in healthy adult cats from the perspectives of growth status, health scores, hematological parameters, serum biochemistry, serum untargeted metabolomics, and gut microbiota. The results showed that 0.2–0.4% LBP supplementation did not significantly affect body weight, ADFI, body condition score, fecal score, or coat score. Hematological and serum biochemical parameters also showed no obvious abnormalities. These findings indicate that the LBP supplementation levels used in this study did not adversely affect the basic health status of adult cats. For adult cats in maintenance, nutritional evaluation should focus not only on body-weight gain but also on metabolic stability and health status [19]. Cats are obligate carnivores with species-specific nutritional metabolism [20], and the effects of functional additives may therefore be reflected more clearly in metabolic homeostasis and gut microbial responses than in growth-related indices.

Hematological and serum biochemical parameters are essential indicators for evaluating animal health and safety. In the present study, white blood cells, red blood cells, hemoglobin, platelets, and other hematological parameters did not differ significantly among groups, suggesting that LBP did not induce obvious inflammation, anemia, or hematological disturbance. Serum biochemical indices, including albumin, total protein, alanine aminotransferase, aspartate aminotransferase, blood urea nitrogen, creatinine, cholesterol, calcium, and inorganic phosphorus, also showed no significant differences, indicating no adverse effects on liver function, renal function, protein metabolism, or routine lipid-related indices. However, routine blood parameters mainly reflect general health status and are relatively insensitive to subtle metabolic changes. Therefore, metabolomics was necessary to further characterize the metabolic effects of LBP.

Differential metabolite analysis showed that all LBP-supplemented groups differed from the control group, but the number and direction of differential metabolites varied among doses. The L group had the largest number of differential metabolites, followed by the M and H groups. The number of differential metabolites should not be simply interpreted as treatment efficacy. A larger number of differential metabolites may indicate a broader metabolic response, whereas fewer metabolites may reflect more focused pathway-level changes. Therefore, the dose effects of LBP should be evaluated by integrating pathway enrichment, key metabolites, and microbiota results.

KEGG enrichment analysis revealed dose-specific metabolic changes. The L group was enriched in fatty acid biosynthesis, BCAA degradation, BCAA biosynthesis, and amino acid biosynthesis, suggesting effects on lipid and amino acid metabolism. Fatty acids are important for membrane structure, energy metabolism, and inflammatory mediator synthesis, and their metabolic changes are closely related to energy homeostasis, immune regulation, and skin and coat health [21]. Dietary lipid components and their metabolites may also affect the intestinal microbiota and gastrointestinal immune homeostasis [22]. Therefore, the pathway changes observed in the L group suggest that low-dose LBP was sufficient to influence basal metabolic status, although further targeted validation is needed to clarify its physiological significance.

In the M group, differential metabolites were mainly enriched in unsaturated fatty acid biosynthesis, with nervonic acid decreasing and linoleic acid increasing. Recent feline nutrition research showed that dietary fat concentration and source can affect fecal microbiota and metabolomic profiles in adult cats, supporting a connection between lipid metabolism and the feline gut microbiome [23]. linoleic acid is an omega-6 polyunsaturated fatty acid and an essential fatty acid in feline diets; it is involved in membrane phospholipid composition, skin barrier maintenance, coat status, and inflammatory and immune responses. The increase in linoleic acid in the M group suggests that medium-dose LBP may mainly participate in unsaturated fatty acid metabolism. Because coat scores did not differ significantly among groups, the metabolic changes observed in this study may not have translated into visible phenotypic differences within the experimental period.

In the H group, enriched pathways included protein digestion and absorption, BCAA degradation, BCAA biosynthesis, aminoacyl-tRNA biosynthesis, lysine degradation, 2-oxocarboxylic acid metabolism, and propanoate metabolism. Key amino acids and peptide-related metabolites, including H-Val-Val-OH, L-leucine, L-valine, L-lysine, and L-pipecolic acid, were downregulated, whereas linoleic acid and 2-methylbutyric acid were upregulated. Amino acids such as leucine, valine, and lysine participate in protein synthesis, energy metabolism, and nutrient signaling [24]. The downregulation of several amino acid-related metabolites in the H group suggests that high-dose LBP may have a stronger influence on amino acid metabolic homeostasis. Importantly, enrichment of the protein digestion and absorption pathway does not prove that LBP promoted or inhibited protein digestion; it only indicates that related metabolites changed. Therefore, the H group results should be interpreted cautiously as changes in amino acid-related metabolic status rather than direct evidence of improved protein digestion.

The key differential metabolite table and clustering heatmap further supported these findings. The distinct expression patterns among the C, L, M, and H groups indicate that different LBP doses induced differential serum metabolite responses. Fatty acid-related metabolites, including palmitoleic acid, decanoic acid, octadecanoic acid, nervonic acid, and linoleic acid, varied among treatment groups, indicating that LBP may modulate lipid metabolism. Amino acid-related metabolites, including L-leucine, L-valine, L-lysine, and L-pipecolic acid, also differed among groups, suggesting simultaneous effects on amino acid metabolism. These results show that the metabolic effects of LBP in adult cats involve multiple metabolic networks rather than a single metabolite or pathway.

Previous feline nutrition studies have shown that resistant starches, fermentable fibers, fiber-bound polyphenols, and changes in dietary lipid source can modify fecal microbial composition, postbiotic production, and metabolite profiles in cats [9,23,25]. Similar to these established nutritional interventions, LBP supplementation in the present study was associated with changes in selected bacterial genera and serum metabolites related mainly to lipid and amino acid metabolism. However, the pattern observed with LBP was not identical to that reported for conventional fibers or prebiotics. LBP is a polysaccharide-rich botanical extract that may contain structurally diverse carbohydrate and non-carbohydrate constituents, whereas many feline prebiotic studies have evaluated more defined fermentable substrates. This compositional complexity may contribute to broader host–microbiota–metabolite responses, but the present experiment did not include a direct functional-fiber or prebiotic comparator. Therefore, the current data do not demonstrate that LBP is superior to established feline functional fibers or prebiotics; instead, they identify LBP as an additional candidate ingredient that warrants direct comparative evaluation. Feline studies of chronic enteropathy further demonstrate associations among intestinal microbial communities, long-chain fatty acids, sterols, and bile acids [26,27], supporting the biological relevance of the microbiota–metabolite relationships observed here.

Beta diversity analysis demonstrated significant differences in microbial community structure between the control group and LBP-supplemented groups, indicating that LBP influenced gut microbial composition. Previous studies have reported that LBP can modulate gut microbiota and glucose-lipid metabolism in animal models [28], attenuate intestinal injury through microbiota regulation [29], and influence host physiology through interactions with gut microbiota and metabolites [30]. These findings support the possibility that modulation of the gut microbiota is an important pathway through which LBP exerts functional effects. Alterations in fecal microbial composition have also been described in cats with intestinal dysbiosis [31].

The absence of a monotonic dose–response pattern suggests that the metabolic and microbial responses to LBP may not increase linearly with supplementation level. The low-dose group showed the broadest metabolite response, the medium-dose group showed comparatively focused changes in unsaturated fatty acid metabolism, and the high-dose group showed stronger changes in amino acid- and protein-related pathways. Such divergence may reflect differences in substrate availability, microbial utilization, host adaptive responses, or threshold effects; however, these mechanisms were not directly tested. The observed genus –metabolite correlations provide a plausible link between microbial restructuring and host lipid or amino acid metabolism, but they remain associative and cannot establish microbial production, metabolite origin, or causality. From a functional pet-nutrition perspective, the present findings identify LBP as a candidate ingredient for further study rather than supporting immediate clinical application or a specific recommended dose. Controlled dose–response trials with larger samples, detailed LBP characterization, repeated sampling, and functional validation are required.

Gut microbiota and host metabolism are closely linked. In this study, Spearman correlation analysis showed associations between key bacterial genera and serum metabolites related to fatty acid and amino acid metabolism. Gut microbiota can participate in host lipid metabolism through effects on lipid absorption, bile acid transformation, and microbial metabolite production [32]. In cats, propionate-producing microbial communities have been associated with body-weight changes in obese cats, suggesting that microbial metabolites may participate in feline energy metabolism [33]. In the present study, *Catenibacterium* was positively correlated with nervonic acid, *Olsenella* was negatively correlated with L-leucine and nervonic acid, *Clostridium* was positively correlated with L-leucine, L-valine, and several fatty acids, and *Peptoclostridium* was positively correlated with palmitoleic acid. These associations suggest that LBP may influence serum lipid and amino acid metabolism through changes in gut microbiota.

Evidence from humans, rodents, poultry, and aquatic animals provides useful mechanistic context for the reported antioxidant, immunomodulatory, metabolic, and microbiota-related effects of LBP [28,29,30,34,35,36]. Nevertheless, these findings cannot be directly extrapolated to domestic cats. Cats are obligate carnivores and differ substantially from humans and rodents in amino acid requirements, hepatic enzyme regulation, lipid utilization, carbohydrate metabolism, gastrointestinal physiology, and gut microbial ecology. Consequently, the biological interpretation of the present results was based primarily on the feline data generated in this study, whereas evidence from other species was used only to support mechanistic plausibility. To strengthen the feline-specific context, the revised Discussion now incorporates published studies of resistant starches, fermentable fibers, fiber-bound polyphenols, dietary lipids, chronic enteropathy, and microbiota-associated metabolites in cats [9,23,25,26,27]. Even with this expanded literature comparison, the present findings remain exploratory and do not establish a feline-specific mechanism or an optimal LBP supplementation level.

This study has several limitations. First, the sample size was relatively small and no formal a priori power analysis was performed; therefore, modest treatment effects may not have been detected, confidence intervals may be wide, and the generalizability of the findings to the broader feline population is limited. Second, cats were assigned using a body-weight-balanced allocation procedure rather than a computer-generated random sequence, and considerable individual variation in initial body weight remained. Third, the nutrient values of the basal diet were obtained from the manufacturer’s guaranteed analysis; metabolizable energy, complete amino acid profiles, and fatty acid profiles were not independently determined. Fourth, although the supplier reported that the commercial LBP product was prepared by water extraction, ethanol precipitation, filtration, concentration, and drying, detailed purification conditions, molecular-weight distribution, monosaccharide composition, HPLC fingerprint, FTIR spectrum, and GPC/HPGPC characterization were unavailable and were not independently determined. This incomplete physicochemical characterization limits reproducibility, prevents attribution of the observed responses to a specific polysaccharide fraction or molecular structure, and means that the findings are specific to the tested batch and should not be generalized to all LBP products. Fifth, although the experimental diets were manually remixed before feeding and no visible powder remained, LBP distribution was not analytically verified. Sixth, different blinded evaluators assessed different score types; although the same evaluator consistently assessed each score type using standardized criteria, some observer-related variation cannot be excluded. Seventh, the M vs. C and H vs. C PLS-DA models had negative Q^2^Y values and limited predictive performance. Eighth, untargeted metabolite annotation relied partly on database matching [37], and some correlations did not remain significant after FDR adjustment. Finally, 16S rRNA gene sequencing describes community composition but does not directly establish microbial function, and correlation analysis cannot establish causality. Larger, independently replicated studies using analytically verified diets, structurally characterized LBP products, targeted metabolomics, and functional microbiome approaches are required.

## 5. Conclusions

Dietary supplementation with nominal LBP levels of approximately 0.2%, 0.3%, or 0.4% did not significantly affect body weight, feed intake, health-related scores, hematological parameters, or serum biochemical indices in healthy adult cats under the present experimental conditions. Exploratory serum metabolomics and 16S rRNA gene sequencing indicated supplementation-associated differences in metabolic signatures and gut microbial composition. Only the L vs. C PLS-DA model showed moderate predictive ability, whereas the M vs. C and H vs. C models had limited predictive performance. Therefore, these findings should be considered preliminary. The study supports further investigation of LBP as a potential functional ingredient in feline diets but does not establish an optimal supplementation level or causal microbiota–metabolite mechanism.

## Figures and Tables

**Table 1:** Manufacturer-declared guaranteed nutrient composition of the basal diet.

Nutrient	Guaranteed Value
Crude protein	≥26.0%
Crude fat	≥10.0%
Crude fiber	≤5.0%
Crude ash	≤10.0%
Moisture	≤10.0%
Calcium	≥0.8%
Total phosphorus	≥0.7%
Taurine	≥0.10%
Water-soluble chloride (as Cl^−^)	≥0.30%

**Table 2:** Growth performance and health scores of adult cats fed diets supplemented with LBP.

Item	C	L	M	H	*p* Value
Initial BW (g)	4021.61 ± 1125.03	3612.57 ± 906.12	3556.22 ± 654.88	3847.87 ± 957.00	0.783
Final BW (g)	4021.67 ± 1237.59	3755.43 ± 813.03	3469.57 ± 530.18	3948.00 ± 969.65	0.689
BW change (g)	0.06 ± 239.94	142.86 ± 234.47	−86.65 ± 255.15	100.13 ± 113.04	0.225
BW change (%)	−0.67 ± 7.29	4.95 ± 8.69	−1.87 ± 6.04	2.61 ± 2.80	0.229
ADFI (g/day)	69.89 ± 13.15	69.92 ± 5.42	68.96 ± 11.00	71.09 ± 8.62	0.982
Body condition score	5.83 ± 0.68	6.00 ± 1.12	5.00 ± 0.50	6.00 ± 0.82	0.122
Fecal score	2.81 ± 0.60	2.71 ± 0.44	2.84 ± 0.55	2.67 ± 0.24	0.907
Coat score	3.17 ± 0.26	3.43 ± 0.53	3.43 ± 0.61	3.07 ± 0.19	0.472

Note: Values are presented as mean ± SD. Effective sample sizes varied among hematological variables because some samples did not yield valid analyzer results; analyses were based on available cases.

**Table 3:** Hematological parameters of adult cats fed diets supplemented with LBP.

Item	C	L	M	H	*p* Value
White blood cells (WBCs, ×10^9^/L)	14.83 ± 2.18	11.83 ± 3.60	13.50 ± 3.71	12.15 ± 4.67	0.533
Neutrophils (NEUTs, %)	62.42 ± 16.09	58.23 ± 13.14	55.00 ± 17.53	61.08 ± 9.29	0.848
Lymphocytes (LYMPHs, %)	27.44 ± 11.38	33.68 ± 15.96	30.37 ± 7.79	31.93 ± 9.68	0.848
Monocytes (MONOs, %)	1.50 ± 0.43	1.93 ± 0.93	2.72 ± 2.35	1.43 ± 0.57	0.438
Eosinophils (EOs, %)	8.40 ± 5.02	5.92 ± 4.98	11.70 ± 12.70	5.47 ± 4.76	0.569
Basophils (BASOs, %)	0.24 ± 0.21	0.23 ± 0.12	0.22 ± 0.15	0.10 ± 0.00	0.464
Neutrophils (NEUT#, ×10^9^/L)	9.42 ± 3.47	7.27 ± 3.76	7.07 ± 2.35	7.74 ± 4.20	0.679
Lymphocytes (LYMPH#, ×10^9^/L)	3.97 ± 1.40	3.60 ± 0.88	4.11 ± 1.63	3.58 ± 0.61	0.866
Monocytes (MONO#, ×10^9^/L)	0.22 ± 0.06	0.22 ± 0.12	0.40 ± 0.41	0.17 ± 0.12	0.441
Eosinophils (EO#, ×10^9^/L)	1.20 ± 0.65	0.71 ± 0.60	1.90 ± 2.58	0.65 ± 0.55	0.493
Basophils (BASO#, ×10^9^/L)	0.03 ± 0.03	0.02 ± 0.01	0.02 ± 0.02	0.01 ± 0.01	0.444
Red blood cells (RBCs, ×10^12^/L)	8.84 ± 0.91	9.08 ± 1.70	7.96 ± 0.41	9.96 ± 1.45	0.119
Hemoglobin (HGB, g/L)	131.40 ± 4.72	138.33 ± 28.17	119.83 ± 3.66	143.75 ± 25.51	0.234
Hematocrit (HCT, %)	39.12 ± 1.45	41.08 ± 8.02	36.35 ± 1.69	43.38 ± 8.45	0.287
Mean corpuscular volume (MCV, fL)	44.58 ± 3.79	45.22 ± 1.85	45.77 ± 3.39	43.33 ± 2.44	0.632
Mean corpuscular hemoglobin (MCH, pg)	14.96 ± 1.20	15.20 ± 0.54	15.08 ± 0.86	14.38 ± 0.59	0.484
Mean corpuscular hemoglobin concentration (MCHC, g/L)	335.20 ± 3.42	336.17 ± 5.95	330.17 ± 11.92	331.75 ± 5.38	0.536
Red cell distribution width (RDW-CV, %)	15.98 ± 0.82	15.73 ± 0.41	15.75 ± 1.00	15.90 ± 0.67	0.941
Red cell distribution width (RDW-SD, fL)	29.98 ± 1.79	29.85 ± 1.68	30.53 ± 3.67	29.00 ± 2.75	0.842
Platelets (PLTs, ×10^9^/L)	223.40 ± 39.37	195.83 ± 44.60	217.67 ± 72.74	240.25 ± 63.85	0.672
Mean platelet volume (MPV, fL)	13.34 ± 0.13	13.37 ± 0.36	13.13 ± 0.34	13.50 ± 0.22	0.284
Platelet distribution width (PDW)	13.96 ± 0.59	13.83 ± 1.36	13.88 ± 0.95	13.22 ± 1.12	0.728
Plateletcrit (PCT, %)	0.30 ± 0.05	0.26 ± 0.06	0.28 ± 0.09	0.32 ± 0.09	0.614

**Table 4:** Serum biochemical parameters of adult cats fed diets supplemented with LBP.

Item	C	L	M	H	*p* Value
Albumin (ALB, g/L)	31.84 ± 1.62	31.08 ± 1.78	31.54 ± 2.56	31.32 ± 3.55	0.908
Total protein (TP, g/L)	82.32 ± 3.94	78.04 ± 4.18	81.12 ± 6.86	80.76 ± 8.11	0.448
Globulin (GLOB, g/L)	50.48 ± 4.22	46.96 ± 5.19	49.58 ± 5.42	49.44 ± 7.53	0.348
Albumin/globulin ratio (ALB/GLOB)	0.63 ± 0.07	0.67 ± 0.10	0.64 ± 0.08	0.64 ± 0.09	0.991
Alanine aminotransferase (ALT, U/L)	69.60 ± 15.01	70.00 ± 20.94	80.20 ± 33.97	61.00 ± 26.76	0.690
Aspartate aminotransferase (AST, U/L)	16.30 ± 4.69	14.60 ± 3.65	18.40 ± 10.90	13.08 ± 7.53	0.690
AST/ALT ratio	0.26 ± 0.13	0.23 ± 0.12	0.21 ± 0.13	0.24 ± 0.18	0.955
Total bilirubin (TBIL, μmol/L)	5.57 ± 0.60	5.54 ± 0.41	4.85 ± 0.51	4.85 ± 0.63	0.083
Alkaline phosphatase (ALKP, U/L)	50.00 ± 26.16	57.60 ± 24.53	34.40 ± 9.99	45.00 ± 9.59	0.315
Blood urea nitrogen (BUN, mmol/L)	7.98 ± 3.71	7.42 ± 1.01	5.97 ± 0.82	6.51 ± 1.41	0.446
Creatinine (CRE, μmol/L)	110.40 ± 30.75	111.40 ± 14.33	89.20 ± 15.64	114.20 ± 24.18	0.296
Inorganic phosphorus (PHOS, mmol/L)	1.49 ± 0.18	1.54 ± 0.21	1.42 ± 0.18	1.53 ± 0.21	0.759
Calcium (Ca, mmol/L)	2.77 ± 0.15	2.69 ± 0.10	2.72 ± 0.09	2.86 ± 0.10	0.151
Total cholesterol (CHOL, mmol/L)	2.53 ± 0.41	2.15 ± 0.41	2.50 ± 0.49	2.76 ± 0.63	0.833
Creatine kinase (CK, U/L)	177.60 ± 204.16	115.00 ± 67.40	62.20 ± 28.82	77.60 ± 52.93	0.394
Amylase (AMY, U/L)	855.20 ± 215.15	974.80 ± 525.01	908.80 ± 316.41	1249.20 ± 447.46	0.424

**Table 5:** PLS-DA model and permutation-test parameters.

Comparison	R^2^Y	Q^2^Y	R^2^ intercept	Q^2^ intercept
L vs. C	0.98	0.40	0.97	−0.74
M vs. C	0.99	−0.26	0.97	−0.40
H vs. C	0.97	−0.01	0.98	−0.41

**Table 6:** Key differential metabolites identified between LBP-supplemented groups and the control group.

Comparison	Ion Mode	Metabolite	FC	*p* Value	FDR	VIP	Trend	Related Pathway
L vs. C	Positive	palmitoleic acid	0.803	0.000576	0.011716	2.705	Down	Fatty acid biosynthesis
L vs. C	Negative	decanoic acid	0.614	0.006501	0.041978	1.867	Down	Fatty acid metabolism
L vs. C	Positive	octadecanoic acid	0.601	0.022539	0.038670	1.781	Down	Fatty acid biosynthesis
L vs. C	Positive	L-leucine	0.817	0.032899	0.043288	1.574	Down	BCAA metabolism
L vs. C	Positive	L-valine	0.774	0.040314	0.045268	1.527	Down	BCAA metabolism
M vs. C	Negative	nervonic acid	0.713	0.018523	0.042783	2.029	Down	Unsaturated fatty acid biosynthesis
M vs. C	Negative	linoleic acid	1.341	0.037305	0.045199	1.705	Up	Unsaturated fatty acid biosynthesis
H vs. C	Positive	H-Val-Val-OH	0.458	0.000187	0.006161	2.493	Down	Protein digestion and BCAA metabolism
H vs. C	Positive	L-leucine	0.809	0.015365	0.034543	2.016	Down	BCAA metabolism
H vs. C	Positive	L-valine	0.753	0.020083	0.038029	2.099	Down	BCAA metabolism
H vs. C	Positive	L-lysine	0.654	0.043079	0.047149	2.062	Down	Protein digestion; Lysine degradation
H vs. C	Positive	L-pipecolic acid	0.776	0.041378	0.046550	2.209	Down	Lysine degradation
H vs. C	Negative	linoleic acid	1.616	0.008195	0.049898	2.528	Up	Fatty acid metabolism
H vs. C	Negative	2-methylbutyric acid	1.679	0.038542	0.049898	2.053	Up	Propanoate metabolism

Note: FC represents the fold change in the experimental group relative to the C group. FDR refers to the *p* value adjusted by the Benjamini–Hochberg method. Up and Down indicate higher and lower relative abundance, respectively, in the experimental group compared with the C group. VIP, variable importance in projection; BCAA, branched-chain amino acids, including valine, leucine, and isoleucine. This table prioritizes differential metabolites related to significantly enriched KEGG pathways and with clear biological relevance.

**Table 7:** ANOSIM analysis of between-group differences in gut microbiota.

Group	R-Value	*p* Value
C-L	0.52037	0.002
C-M	0.67222	0.005
C-H	0.27037	0.043

## Data Availability

The data presented in this study are available from the corresponding author upon reasonable request.
